# Exploring the impact of sense of work gain on kindergarten teachers’ work performance: the mediating role of organizational commitment and the moderating influence of supportive leadership

**DOI:** 10.3389/fpsyg.2025.1515054

**Published:** 2025-04-01

**Authors:** Wenxiu Shi, Yile Zheng

**Affiliations:** School of Education, Baoji University of Arts and Sciences, Baoji, China

**Keywords:** kindergarten teachers, sense of work gain, job performance, organizational commitment, supportive leadership

## Abstract

**Introduction:**

The concept of sense of work gain encapsulates the profound psychological gratification and forward-looking aspirations that arise when an individual’s dedicated efforts at work are met with equitable material and spiritual compensation. Despite a wealth of research on its correlation with job satisfaction and innovative behaviors among educators, the underlying mechanisms linking sense of work gain to job performance remain understudied. This study introduces a nuanced moderated mediation framework to elucidate the pathways through which sense of work gain influences the job performance of teachers, spotlighting the pivotal role of organizational commitment and the nuanced impact of supportive leadership.

**Methods:**

With a focus on the kindergarten teaching workforce, an extensive survey was administered to 1,081 educators across Shaanxi and Gansu provinces in China. Subsequent hypothesis testing was performed using statistical tools SPSS 27.0 and AMOS 24.0.

**Results:**

The research results indicated that sense of work gain had a significant positive predictive effect on the job performance of kindergarten teachers. Organizational commitment played a mediating role between sense of work gain and the job performance of kindergarten teachers. Supportive leadership not only positively moderated the relationship between sense of work gain and the job performance of kindergarten teachers but also positively moderated the relationships between sense of work gain and organizational commitment, as well as between organizational commitment and job performance.

**Discussion:**

These findings revealed a significant positive effect of job fulfillment on the work performance of kindergarten teachers and elucidated the mediating effect of organizational commitment as well as the positive moderating role of supportive leadership in this process. These discoveries emphasized the importance of enhancing teachers’ job fulfillment, strengthening organizational commitment, and cultivating a supportive leadership style for optimizing the work performance of kindergarten teachers, providing education administrators with effective strategies for improving teachers’ working environment and enhancing teaching quality.

## 1 Introduction

Teachers’ Job performance (JP) is instrumental not only in ensuring the quality of education but also in gauging their professional development trajectory (Šimunović et al., 2023). Empirical evidence underscores the profound impact of teachers’ JP on students’ learning outcomes and personal development (Yao et al., 2024). In the formative years of kindergarten education, the role of teachers’ JP becomes paramount in nurturing students’ early learning abilities and social adaptability (Han, 2008). Consequently, a profound examination and comprehension of the multifaceted factors that influence teachers’ JP are crucial for elevating educational standards and catalyzing teachers’ professional progression.

Despite the myriad factors that shape JP, including the work environment (Li T. et al., 2024), organizational support, and emotional commitment (Lindner et al., 2021), the latent influence of sense of work gain (SWG), an intrinsic motivational force, on JP has been the subject of limited scholarly inquiry. Rooted in Self-Determination Theory (Deci and Ryan, 2000), SWG reflects the fulfillment of teachers’ intrinsic psychological needs—autonomy, competence, and relatedness—through personal growth, recognition, and accomplishment derived from their professional endeavors (Khan, 2023). This intrinsic motivation mechanism is posited to enhance job engagement and subsequent JP by fostering volitional persistence and goal-directed behavior. Nonetheless, the current scholarly corpus is notably deficient in research examining the intricate relationship between SWG and teachers’ JP, thereby presenting a compelling avenue for exploration in this study.

The existing body of research underscores the substantial positive impact of the SWG on various aspects of teachers’ experiences, including job satisfaction (Boštjančič and Petrovčič, 2019), professional commitment (Shi et al., 2023), and the propensity for innovation (Gu et al., 2020). Despite this, the majority of studies have concentrated on the direct effects of the SWG, with scant attention paid to its indirect influence on JP through mediating factors. Guided by social exchange theory (Blau, 1964), we propose that SWG fosters organizational commitment (OC) through reciprocal dynamics: when teachers perceive equitable gains (e.g., recognition, skill development) from their work, they reciprocate with heightened loyalty and discretionary effort, which in turn enhances JP. Additionally, while it has been posited that the beneficial effects of the SWG might be predicated on conditions such as organizational commitment (OC) (Zheng, 2022), the nuanced mechanisms at play require further elucidation. This study endeavors to fill this void by conducting a comprehensive examination of the influence of the SWG on the JP of preschool teachers, as well as the mechanisms involved.

By centering on preschool educators, this study delves into the ramifications of the SWG on JP, while also scrutinizing the mediating influence of OC and the moderating impact of supportive leadership (SL). From the perspective of conservation of resources theory (Hobfoll, 1989), supportive leadership acts as a boundary condition that mitigates resource depletion, enabling teachers to effectively translate SWG into performance-enhancing behaviors. The findings not only contribute to a more profound understanding of the SWG’s role in shaping JP but also present actionable strategies for educational leaders to bolster teachers’ SWG, thereby enhancing their JP.

## 2 Literature review and hypotheses development

### 2.1 SWG and JP

The concept of SWG encapsulates a localized connotation within China’s specific cultural context, emphasizing the sense of achievement and satisfaction that individuals experience in their work environment (Cui and Li, 2023). For kindergarten teachers, the SWG manifests not only in explicit gains at the material level, such as salary increases and improved welfare benefits, but also in implicit gains at the spiritual level, such as enhanced professional identity, satisfaction derived from professional growth, and emotional connections gained through interactions with children and parents (Huang and Zhou, 2024).

As a concept in organizational behavior, JP generally refers to the comprehensive embodiment of the quantity, quality, efficiency, and outcomes of work completed by an individual within a certain period (Op't Eynde et al., 2023). For kindergarten teachers, JP is reflected not only in the effectiveness and innovation of daily teaching and care work but also involves aspects of management performance and relational performance, such as class management, home-school communication, and children’s emotional and social development. High-performing kindergarten teachers can promote the comprehensive development of children and create a good educational atmosphere for the kindergarten, enhancing the overall quality of education (Valdez and Mendoza, 2024).

According to Self-Determination Theory (Deci and Ryan, 2000), the satisfaction of individuals’ intrinsic motivation and autonomy needs is a key factor influencing their behavioral performance. As a specific embodiment of teachers’ intrinsic motivation, the SWG can significantly promote the improvement of their JP. The SWG can enhance kindergarten teachers’ self-efficacy and psychological resilience, enabling teachers to calmly cope with challenges and pressures in their work and improve relational performance, such as strengthening home-school communication and promoting team collaboration (Li Y. et al., 2024). Existing research also indicates that the SWG has a significant positive impact on teachers’ job satisfaction (Boštjančič and Petrovčič, 2019), OC and work engagement (Huang and Zhou, 2024). These positive work attitudes and behavioral changes ultimately translate into improvements in JP. Therefore, we propose:

*Hypothesis 1*: The SWG has a significant positive predictive effect on the JP of kindergarten teachers.

### 2.2 OC as a mediating variable

Organizational commitment is characterized by an individual’s alignment with and allegiance to a specific organization and its objectives, representing an essential psychological nexus that unites the individual with the organization (Meyer and Allen, 1991). High OC among kindergarten teachers not only contributes to enhancing the quality of education in kindergartens but also strengthens teachers’ professional stability and sense of happiness (Li, 2023). Importantly, empirical evidence has consistently shown that OC is a pivotal predictor of various teacher outcomes, including job satisfaction, work engagement, and career advancement (Jing and Zhang, 2014), which are integral to the sustenance and vitality of the educational community within kindergartens.

Social exchange theory (Blau, 1964) posits that the dynamic between employees and their organizations is inherently transactional in nature. Employees who perceive organizational support and validation, thereby augmenting their SWG, are more likely to cultivate a favorable organizational assessment and a deep-seated emotional bond, which in turn, escalates their OC (Rhoades and Eisenberger, 2002). Furthermore, teachers with elevated levels of OC are inclined to integrate their career aspirations with the kindergarten’s collective aspirations, approaching their duties with heightened fervor and a profound sense of responsibility, thus augmenting JP (Li, 2023). In light of this, we propose:

*Hypothesis 2*: OC serves as a mediating variable in the relationship between the SWG and the JP of kindergarten teachers.

### 2.3 SL as a moderator

Supportive leadership is a management style that emphasizes leaders providing emotional, resource, and developmental support to their subordinates (Bass, 1985). This comprehensive support encompasses attending to personal well-being, empowering autonomous decision-making, and furnishing essential resources and feedback (Bass and Avolio, 1994). Research underscores the significant role of SL in elevating teachers’ job satisfaction, fortifying their professional identity and igniting their work enthusiasm and innovation (Liu et al., 2021).

Grounded in Social Exchange Theory (Blau, 1964), employees are inclined to reciprocate with positivity when they perceive organizational support. The SWG, an individual’s subjective evaluation of rewards stemming from their work endeavors, wields a profound influence on kindergarten teachers’ JP (Gu et al., 2020). Empirical evidence attests to the positive moderating effect of SL on the relationship between job satisfaction and JP (Khalid et al., 2012), suggesting that in SL environments, teachers’ positive sentiments toward work are more likely to manifest in exceptional performance. Hence, we posit the following hypothesis:

*Hypothesis 3*: SL positively moderates the relationship between the SWG and JP among kindergarten teachers.

Research indicated that SL can enhance employees’ sense of organizational belonging and job satisfaction (Eisenberger et al., 1986). Notably, SWG, embodying teachers’ positive perceptions of their job roles and surroundings, has been empirically validated to bolster job satisfaction and professional commitment (Buonomo et al., 2023). Moreover, SL not only directly elevates teachers’ OC through caring and empowering practices, but also acts as a catalyst, facilitating the transformation of SWG into robust OC (Liu et al., 2021). Drawing upon this corpus of research, we posit:

*Hypothesis 4*: SL exerts a positive moderating influence on the relationship between SWG and OC among kindergarten teachers.

In accordance with the Organizational Support Theory (Eisenberger et al., 1986), SL potently nurtures teachers’ professional identity and work motivation, which subsequently cascades into enhanced JP. By embodying care, trust, and respect, SL fosters a conducive work environment (Rafferty and Griffin, 2006). When teachers feel strong support from their leaders, their OC will translate into a more positive work attitude and behavior, thereby enhancing their JP (Buluc and Gunes, 2014). Essentially, in environments characterized by SL, the positive correlation between teachers’ OC and JP becomes even more pronounced. Consequently, we propose:

*Hypothesis 5*: SL exerts a positive moderating influence on the relationship between OC and JP among kindergarten teachers.

**Figure 1:**
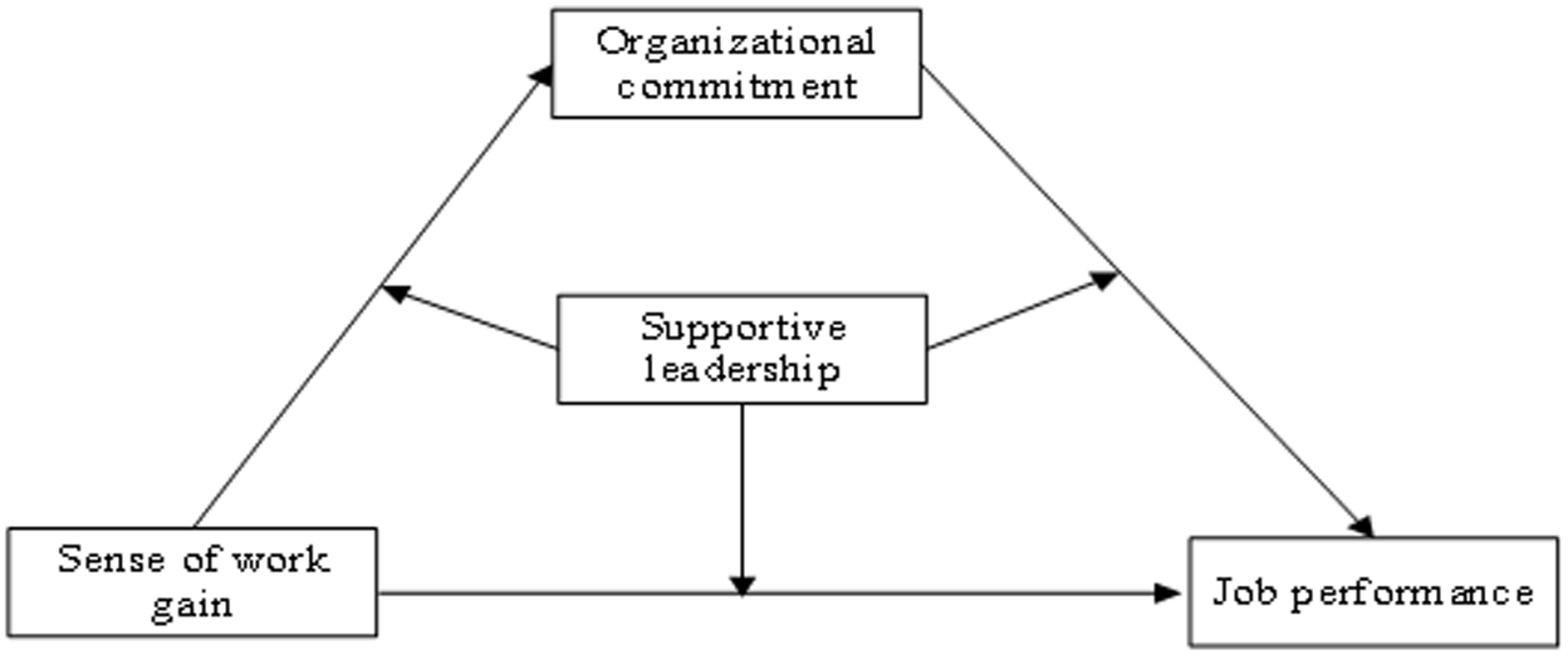
Theoretical model.

Based on the above analysis, we have constructed the theoretical model for this study (as shown in Figure 1).

## 3 Methods

### 3.1 Participants

This study employed a convenience sampling strategy to recruit kindergarten teachers from 52 kindergartens in Shaanxi and Gansu provinces, China. Data were collected between March and April 2024 through a combination of online surveys (Wenjuanxing platform) and on-site visits. A total of 1,081 questionnaires were distributed, with 916 returned (initial response rate: 84.7%). After excluding invalid responses (e.g., patterned answering, missing demographic data), 843 valid questionnaires were retained, yielding an effective response rate of 77.98% (843/1,081).

To assess non-response bias, we followed Armstrong and Overton’s (1977) method by comparing early responders (collected within 2 weeks, *n* = 632) and late responders (collected in weeks 3–4, *n* = 211) on key variables (sense of work gain, organizational commitment) using independent t-tests. No significant differences were found: sense of work gain (*t* = 0.94, *p* = 0.346) and organizational commitment (*t* = 1.11, *p* = 0.268), indicating minimal non-response bias.

### 3.2 Variables

#### 3.2.1 Independent variable

The independent variable, SWG, was operationalized using the Employee Work Gain Scale (Zhu and Liu, 2020), which includes 13 items across four dimensions: workplace dignity, remuneration satisfaction, capability development, and professional aspirations. Prior to CFA, we conducted exploratory factor analysis (EFA) using principal component analysis (PCA) with Varimax rotation on a randomly split subsample (*n* = 421). The Kaiser–Meyer–Olkin (KMO) measure of sampling adequacy was 0.925 (>0.80), and Bartlett’s test of sphericity was significant (*χ*^2^ = 5,184.588, *p* < 0.001), confirming factorability. PCA extracted four components explaining 84.789% of the total variance, with all items loading >0.60 on their respective dimensions. Subsequent CFA on the full sample confirmed strong model fit (*χ*^2^/df = 2.448, RMSEA = 0.041, CFI = 0.988).

#### 3.2.2 Dependent variable

Job performance (JP) was measured using Williams and Anderson’s (1991) 4-item scale. EFA (KMO = 0.842, Bartlett’s *χ*^2^ = 1133.912, *p* < 0.001) revealed a single-factor structure explaining 78.871% variance, with item loadings ranging from 0.881 to 0.907. In the current study, the Cronbach’s *α* coefficient for the Job Performance Scale surpassed the acceptable threshold of 0.7, reaching 0.831. CFA further validated the unidimensional structure (*χ*^2^/df = 3.075, RMSEA = 0.050, CFI = 0.997).

#### 3.2.3 Mediating variable

Organizational commitment (OC) was assessed via Meyer and Allen’s (1991) 18-item OCQ. EFA (subsample *n* = 421, KMO = 0.960, Bartlett’s *χ*^2^ = 7996.243, *p* < 0.001) supported a three-factor solution (affective, continuance, normative commitment), explaining 76.925% variance. The OCQ exhibited an overall Cronbach’s α coefficient of 0.942 in our study, with individual Cronbach’s α coefficients for the aforementioned dimensions standing at 0.885, 0.881, and 0.870. CFA confirmed the tripartite structure (*χ*^2^/df = 4.148, RMSEA = 0.061, CFI = 0.958).

#### 3.2.4 Moderating variable

Supportive leadership (SL) was measured using Rooney and Gottlieb’s (2007) 8-item scale. EFA (KMO = 0.951, Bartlett’s *χ*^2^ = 4,591.809, *p* < 0.001) identified a single factor explaining 84.589% variance, with loadings between 0.898 and 0.947. In this study, the Cronbach’s α coefficient for this scale was found to be 0.894, indicating high reliability. CFA demonstrated excellent fit (*χ*^2^/df = 1.968, RMSEA = 0.034, CFI = 0.995).

#### 3.2.5 Control variable

Drawing upon a robust body of prior research, several demographic factors were identified as potential influencers of preschool teachers’ psychological well-being and subsequent JP. These factors encompass teachers’ gender, educational attainment, years of teaching experience, professional discipline, and the unique characteristics of the kindergarten environment (Wu et al., 2011). In light of these findings, we have meticulously incorporated these five demographic variables as control variables within the ambit of our study.

## 4 Statistical analysis

The statistical analysis encompasses three components: common method bias testing, descriptive statistics and correlation analysis, and hypothesis testing. For assessing common method bias, we utilized Harman’s single-factor test alongside confirmatory factor analysis (CFA) that incorporated a common method factor. Hypothesis testing was conducted using the PROCESS macro (Version 4.0; Hayes, 2018) in SPSS 27.0, a regression-based approach specifically designed for testing mediation, moderation, and conditional process models.

To address the research questions, we implemented a two-step analytical approach: First, Model 4 with 5,000 bootstrap resamples tested the indirect effect of SWG on JP through OC. Second, Model 59 examined supportive leadership (SL) as a moderator of both the SWG → OC and OC → JP pathways. This approach aligns with recommendations for testing conditional indirect effects in regression frameworks (Edwards and Lambert, 2007). Variables were mean-centered to mitigate multicollinearity in interaction terms (Aiken and West, 1991).

The regression-based PROCESS approach was selected for three key reasons. First, it aligns theoretically with our focus on mediation and moderated mediation effects, as it directly tests these hypotheses without requiring complex latent variable modeling. Second, bootstrapping with 5,000 resamples ensures statistical robustness by generating bias-corrected confidence intervals for indirect effects, effectively addressing non-normality common in social science data. Finally, PROCESS enhances methodological transparency through its specialized design for conditional process analysis, allowing simultaneous examination of supportive leadership’s moderating effects across multiple pathways (Hayes, 2018).

### 4.1 Common method bias

This study implemented two methods to examine the potential influence of common method bias. Initially, Harman’s single-factor test was utilized to assess common method bias. All measurement items were aggregated into an exploratory factor analysis, which revealed that the maximum factor accounted for 30.514% of the variance in the unrotated factor analysis results. According to Zhou and Long (2004), a maximum factor variance below 40% suggests that significant common method bias is not present. Additionally, we employed Amos 24.0 to conduct confirmatory factor analysis, assessing the validity of the measurement variables. The theoretical model for this study includes four factors: SWG, JP, OC, and SL. We established three alternative models for comparison: a three-factor model (SWG + OC, SL, and JP), a two-factor model (SWG + OC + SL, JP), and a single-factor model (SWG + OC + SL + JP). The results indicated that fit indices for the four-factor model (*χ*^2^/df = 2.752, RMSEA = 0.046, SRMR = 0.031, CFI = 0.968, TLI = 0.962) significantly outperformed those of the other models (refer to Table 1).

**Table 1:** Confirmatory factor analysis of variables.

Model	χ^2^	*df*	χ^2^/*df*	∆χ^2^(*df*)	CFI	TLI	RMSEA	SRMR
4-factor	401.722	146	2.752		0.968	0.962	0.046	0.031
3-factor	1,485.768	149	9.972	1,084.046 (3)***	0.831	0.806	0.103	0.078
2-factor	3,162.548	151	20.944	2,760.826 (5)***	0.620	0.569	0.154	0.144
1-factor	4,201.864	152	27.644	3,800.142 (6)***	0.489	0.425	0.178	0.154

### 4.2 Descriptive statistics and correlation analysis

Table 2 presents the mean, standard deviation, and correlation coefficients of all variables. It is evident that there is a significant positive correlation between the SWG and both OC (*r* = 0.421, *p* < 0.01) and JP (*r* = 0.383, *p* < 0.01). Furthermore, a significant positive correlation also exists between OC and JP (*r* = 0.406, *p* < 0.01). These correlations provide a preliminary foundation for the subsequent hypothesis testing.

**Table 2:** Means, standard deviations, and correlations (*N* = 843).

Variable	1	2	3	4	5	6	7	8	9
1. Gender	1								
2. Education level	−0.02	1							
3. Teaching experience	0.02	0.10**	1						
4. Professional background	−0.14**	−0.02	0.04	1					
5. Kindergarten type	0.07*	−0.28**	−0.17**	−0.01	1				
6. SWG	−0.05	−0.15**	0.11**	0.01	−0.01	1			
7. OC	−0.06	−0.06	0.02	0.01	0.04	0.42**	1		
8. SL	−0.04	−0.05	−0.02	0.00	−0.02	0.24**	0.32**	1	
9. JP	−0.03	−0.04	0.04	−0.06	−0.06	0.38**	0.41**	0.27**	1
*M*	0.96	2.68	1.79	0.27	0.25	3.93	3.68	3.85	3.82
SD	0.21	0.63	1.01	0.44	0.43	0.68	0.74	0.78	0.72

*p < 0.05, **p < 0.01.

### 4.3 Hypotheses testing

Initially, we standardized our variables and employed Hayes’ (2017) PROCESS macro Model 4 to rigorously examine the mediating mechanism of OC. Here, SWG served as the independent variable, JP as the dependent variable, and OC as the mediator, with controls including gender, educational background, teaching experience, professional discipline, and kindergarten type. As demonstrated in Table 3, a robust and statistically significant positive relationship emerges between SWG and JP (*β* = 0.38, *p* < 0.001), thereby upholding Hypothesis 1. Notably, even after accounting for the mediating variable, the positive influence of SWG on JP remains substantial (*β* = 0.26, *p* < 0.001). Furthermore, both the effect of SWG on OC (*β* = 0.42, *p* < 0.001) and that of OC on JP (*β* = 0.30, *p* < 0.001) are markedly positive, conclusively validating the significant mediating role of OC between these constructs, as per Hypothesis 2. A closer inspection of Table 4 reveals that the mediating effect of OC contributes 0.12 to the overall effect, with a 95% confidence interval ranging from 0.09 to 0.16, exclusive of zero, signifying a noteworthy 31.58% share in the total impact.

**Table 3:** Analysis of the mediating effect of OC.

Regression equation		Fit indicators			Coefficient significance	
Outcome variable	Predictor variable	*R*	*R^2^*	*F*	*β*	*t*
JP		0.39	0.15	25.40***		
	SWG				0.38	11.83***
OC		0.43	0.18	30.88***		
	SWG				0.42	13.29***
JP		0.48	0.23	35.28***		
	SWG				0.26	7.51***
	OC				0.30	8.95***

In the regression analysis, gender, educational attainment, teaching experience, professional background, and kindergarten type were all treated as control variables.*p < 0.05, **p < 0.01, ***p < 0.001.

**Table 4:** Decomposition of total, direct, and mediation effects.

Effect type	Effect value	Standard error	95% CI		Effect proportion
			Lower limit	Upper limit	
Total effect	0.38	0.03	0.32	0.45	
Direct effect	0.26	0.03	0.19	0.32	68.42%
Mediated effect of OC	0.12	0.02	0.09	0.16	31.58%

**Figure 2:**
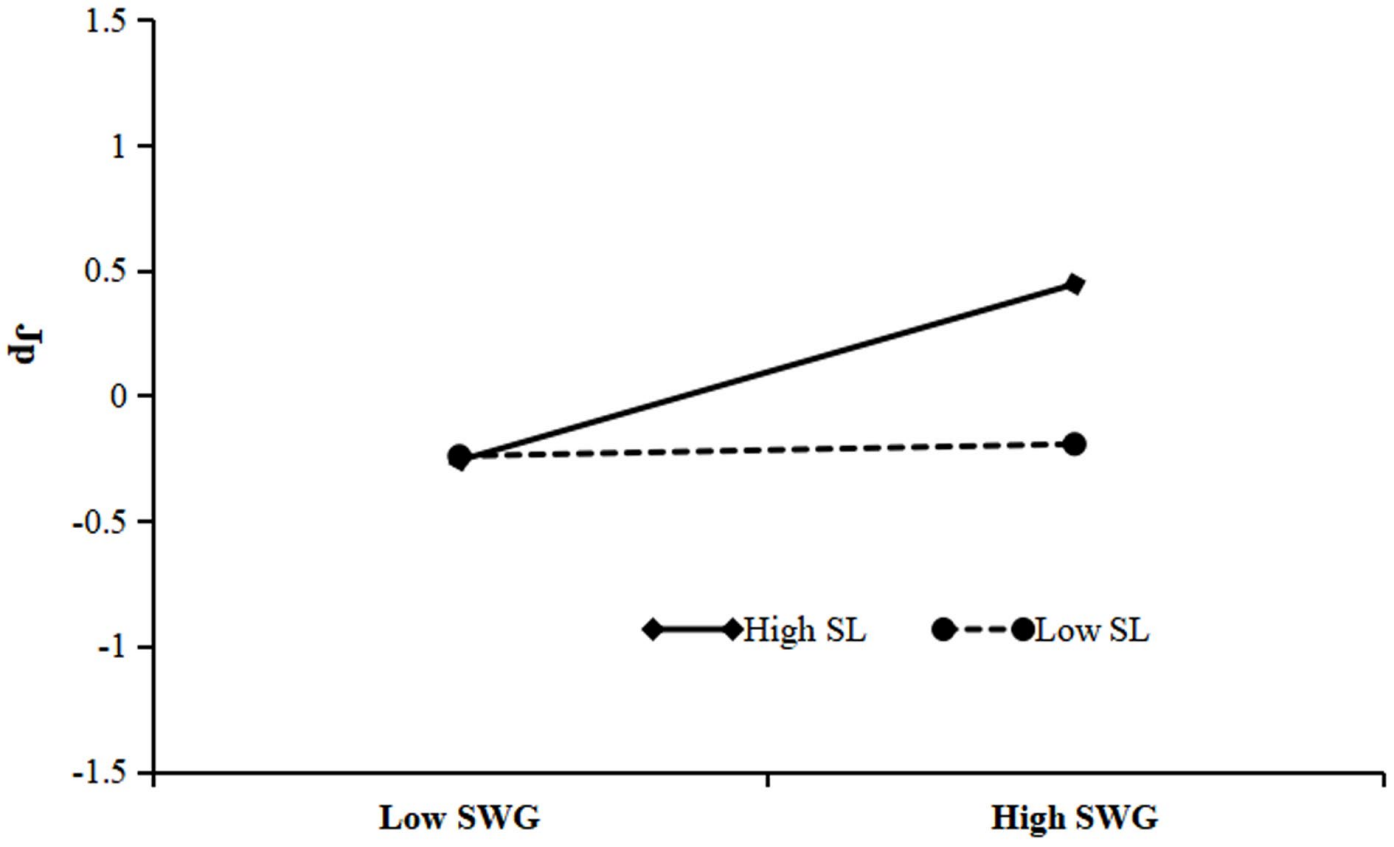
Moderating effect of SL on the relationship between SWG and JP.

Incorporating SL as a moderating factor, we conducted a moderated mediation analysis utilizing the PROCESS macro model 59, as detailed in Table 5. Our findings underscore a significant and positive interaction effect between the SWG and SL on JP (*β* = 0.16, *p* < 0.001). Visualized in Figure 2, the interaction plot and subsequent simple slope tests elucidate that when SL is low, the SWG fails to significantly impact JP (*β* = 0.02, *p* > 0.05). Conversely, under conditions of heightened SL, the SWG exerts a marked and positive influence on JP (*β* = 0.35, *p* < 0.001). Therefore, SL exerts a positive moderating effect between SWG and JP. Hypothesis H3 is confirmed.

**Table 5:** Analysis of moderating effects.

Variable	OC		JP	
	*β*	*t*	*β*	*t*
SWG	0.32	9.69***	0.19	5.51***
OC			0.23	6.72***
SL	0.25	8.07***	0.16	4.90***
SWG*SL	0.17	5.44***	0.16	4.46***
OC*SL			0.07	2.35*
*R*	0.51		0.53	
*R* ^2^	0.26		0.28	
*F*	36.31***		32.88***	

*p < 0.05, **p < 0.01, ***p < 0.001.

**Figure 3:**
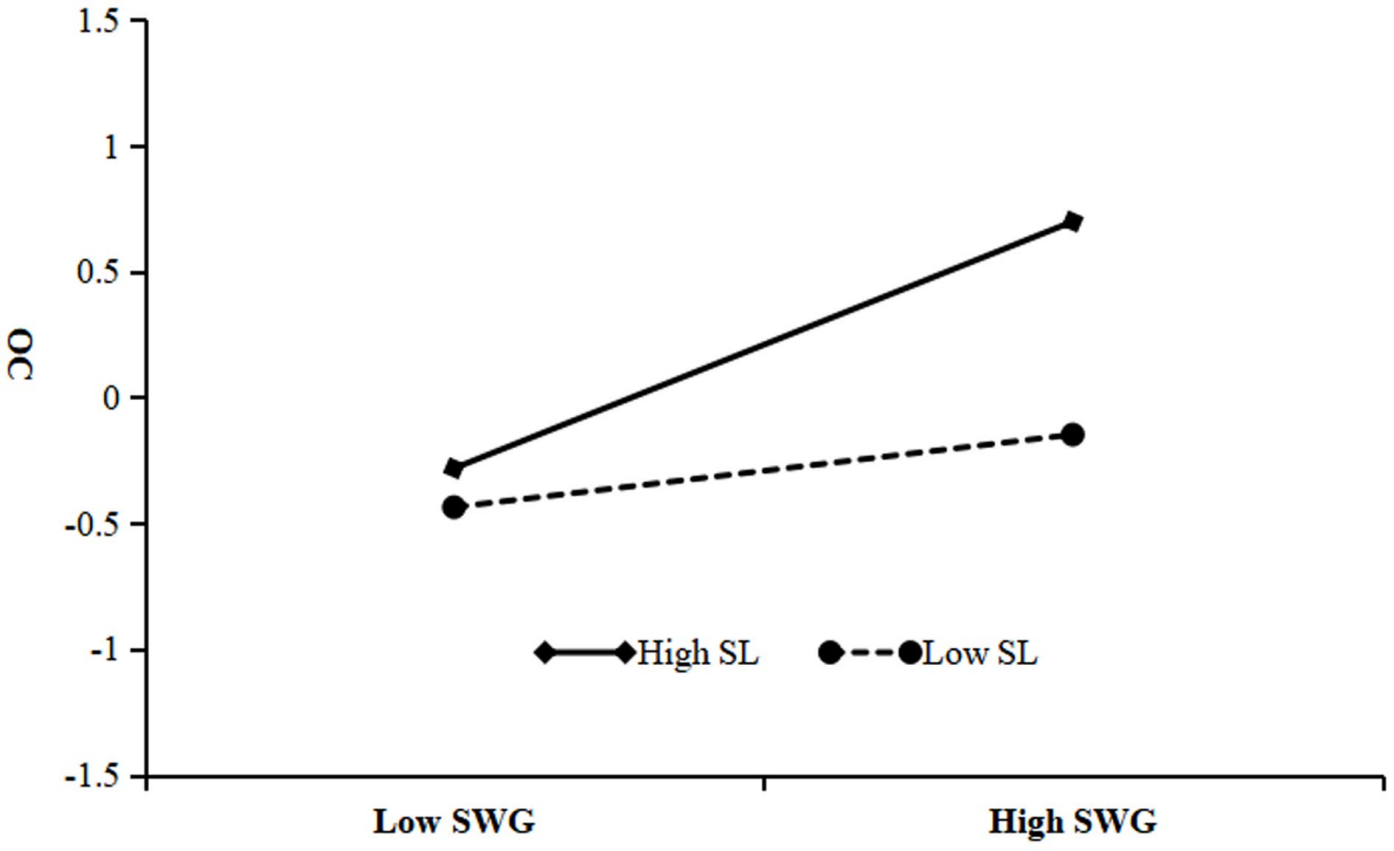
Moderating effect of SL on the relationship between SWG and OC.

**Figure 4:**
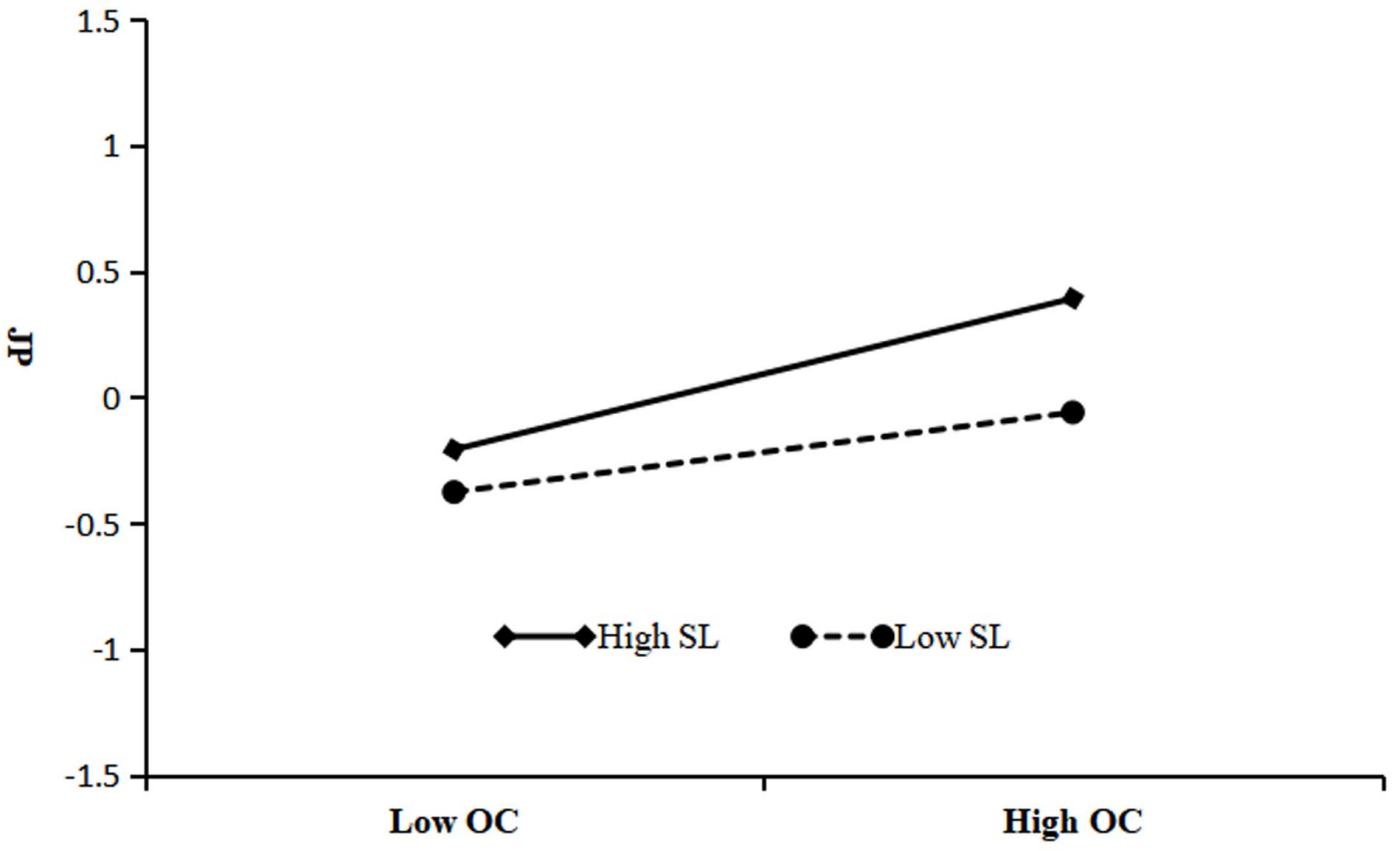
Moderating effect of SL on the relationship between OC and JP.

Table 5 delineates that the interplay between SWG and SL robustly fosters OC (*β* = 0.17, *p* < 0.001). Referencing Figure 3, the interaction effects and simple slopes suggest that at subdued levels of SL, SWG imparts a significant, albeit moderate, positive influence on OC (*β* = 0.14, *p* < 0.01). Conversely, at elevated SL levels, this influence markedly intensifies (*β* = 0.49, *p* < 0.001), corroborating that SL acts as an effective moderator within the nexus of SWG and OC, thereby validating Hypothesis 4. Moreover, Table 5 also illustrates that the synergy between OC and SL significantly propels JP (*β* = 0.07, *p* < 0.05). As depicted in Figure 4, under lesser SL, OC has a discernible positive impact on JP (*β* = 0.16, *p* < 0.001); whereas, at higher levels, the SWG profoundly augments JP (*β* = 0.30, *p* < 0.001), confirming that SL constructively regulates the interrelation between OC and JP, affirming Hypothesis 5.

## 5 Discussion

### 5.1 The relationship between SWG and JP of preschool teachers

This study underscores a salient positive predictive relationship between preschool educators’ SWG and their JP, substantiating Hypothesis 1 and echoing the tenets of Self-Determination Theory and Social Exchange Theory (Boštjančič and Petrovčič, 2019). The theory posits that when educators perceive both intrinsic fulfillment and extrinsic support in their work, they exhibit heightened motivation and superior performance (Blau, 1964). A robust SWG signifies that preschool educators find a sense of achievement, recognition, and belonging in their profession, fostering a positive psychological stance that ignites passion, sparks creativity, and ultimately elevates teaching standards and operational efficacy (Bakker et al., 2010). However, alternative explanations for this relationship warrant consideration. For instance, teachers’ intrinsic personality traits (e.g., conscientiousness) or external resource availability (e.g., classroom materials) might confound the SWG-JP link, as these factors independently influence both perceived work gains and performance outcomes (Podolny et al., 2004). Amidst the burgeoning landscape of preschool education, educators confront obstacles to optimizing JP due to occupational stress and inadequate support structures, culminating in a diminished SWG (Dogan and Çelik, 2019). Consequently, to augment preschool educators’ JP, kindergarten administrators must prioritize fostering their SWG.

### 5.2 The mediation of OC

Our research underscores the pivotal role of OC in mediating the relationship between preschool teachers’ SWG and their JP, thereby validating Hypothesis 2. This finding aligns seamlessly with the tenets of Self-Determination Theory and Social Exchange Theory (Blau, 1964), underscoring that when preschool educators derive profound satisfaction from their work, they are more likely to develop a profound commitment to their organization, which in turn bolsters their JP. Our investigation delves deeper, revealing that as preschool teachers’ SWG intensifies, they become more vested in contributing to the organization’s aspirations and objectives. This positive work ethic and behavior culminate in the enhancement of their JP. Consequently, kindergarten administrators must prioritize nurturing teachers’ OC by fostering a vibrant organizational culture that fosters a profound sense of belonging and organizational identification, thereby solidifying and advancing their commitment (Dogan and Çelik, 2019). While our findings emphasize OC’s mediating role, other unmeasured variables could partially explain the SWG-JP relationship. For example, work engagement or emotional intelligence might act as parallel mediators, as they similarly channel resource gains into performance (Boštjančič and Petrovčič, 2019). Additionally, potential confounding variables such as teachers’ years of experience or kindergarten size were not controlled, which may influence both SWG and OC (Buluc and Gunes, 2014). Future studies should incorporate these factors to isolate OC’s unique contribution.

### 5.3 The moderating role of SL

This study delves into the intricate interplay between SL and the SWG, exploring how this dynamic duo modulates preschool teachers’ JP. SL characterized by emotional backing, achievement recognition, and a focus on individual growth, fosters employee motivation and job satisfaction (Cheng and Couture, 2000). Prevailing research underscores the pivotal role of SL in bolstering JP and deepening OC (Shi et al., 2023). In harmony with this body of work, our findings affirm that the salutary influence of the SWG on preschool teachers’ JP is intricately moderated by SL. Theoretically, our study offers empirical grounding for the moderating mechanism of SL within the nexus of SWG and JP, thereby enriching the theoretical tapestry surrounding teachers’ work motivations and outcomes. Furthermore, it sheds light on the moderating influence of SL in the relationship between OC and JP, emphasizing the paramount significance of leadership style in sculpting employees’ attitudes and behaviors (Bai et al., 2020). Consequently, kindergarten administrators ought to prioritize embodying SL practices by actively engaging with teachers, addressing their developmental needs, and providing the necessary resources and emotional support to amplify their SWG and OC, thereby elevating JP. It is important to acknowledge that alternative moderators, such as organizational climate or policy support systems, might interact with SL to shape the SWG-OC-JP pathways. For instance, in kindergartens with robust institutional resources, SL’s moderating effects could be less pronounced, as structural support compensates for leadership variability (Wu et al., 2011). Furthermore, workload intensity—a variable not directly measured here—might confound these relationships; teachers with lighter workloads may perceive stronger SWG regardless of SL (Bass, 1985). We encourage future research to disentangle these dynamics using multi-level designs.

## 6 Conclusion, suggestions and limitations

This study meticulously examines the intricate mechanisms by which the SWG influences preschool teachers’ JP, while also elucidating the pivotal mediating role of OC and the salient moderating influence of SL. The results conclusively show that the SWG not only serves as a significant positive predictor of preschool teachers’ JP but also partially transmits this positive effect through the mediation of OC. Furthermore, SL emerges as a positive moderator, refining the relationship between the SWG and both JP and OC, underscoring the paramount importance of leadership style in fostering enriching work experiences and fostering a sense of commitment among teachers. Notably, SL also amplifies the relationship between OC and JP, emphasizing the transformative power of a nurturing leadership environment in boosting JP. These revelations not only enrich the theoretical framework linking SWG, OC, SL, and JP but also offer actionable guidance for kindergarten administrators striving to elevate teachers’ work efficacy.

Drawing from the research insights outlined above, this manuscript advances a suite of targeted recommendations: Initially, it is essential for kindergartens to elevate the SWG among their teaching staff by instituting professional training programs, equitable remuneration packages, and clear career progression pathways to bolster their professional gratification and work impetus. Subsequently, the cultivation of a SL milieu by kindergarten administrators is paramount, characterized by open dialog with teachers and the provision of empathetic support to galvanize a robust sense of OC and active job engagement. Additionally, the nurturing of a vibrant organizational culture and a nurturing work ambiance is crucial to instilling in teachers a profound sense of warmth and belonging, which can significantly augment their JP. Finally, the institution of a responsive feedback mechanism is advised, designed to efficiently assimilate the insights and recommendations of the teaching staff, facilitating ongoing enhancements in administrative practices and service delivery to progressively elevate the job contentment and performance metrics of the educators.

This study is not without limitations. Firstly, due to its reliance on cross-sectional survey data, it faces difficulties in definitively establishing causal relationships between variables (Lindner et al., 2021). To gain deeper insights, future research should adopt a longitudinal design that allows for a more nuanced exploration of the dynamic and causal relationships among these variables. Secondly, the sample for this study, primarily consisting of kindergarten teachers from Shaanxi and Gansu provinces in mainland China, may limit the universality of the findings. To enhance the generalization and representativeness of the conclusions, future research should broaden its scope and collect data from a national or even international level. Lastly, the reliance on teacher self-reports for data collection may introduce subjective biases. To improve data objectivity and accuracy, future studies could consider incorporating feedback from multiple sources, such as principals, colleagues, or parents.

## Data Availability

The raw data supporting the conclusions of this article will be made available by the authors, without undue reservation.
